# Dietary supplementation with mushroom powder (*Agaricus bisporus*) on performance, carcass traits, meat quality, and bone biomechanical properties of quail (*Coturnix coturnix japonica*)

**DOI:** 10.1007/s11250-024-03920-4

**Published:** 2024-02-15

**Authors:** Seyit Ahmet Gökmen, Kübra Ünal, Osman Olgun, Behlül Sevim, Ainhoa Sarmiento-García

**Affiliations:** 1 Department of Animal Science, Agriculture Faculty, Selcuk University, 42130 Konya, Türkiye; 2 Department of Food Engineering, Agriculture Faculty, Selcuk University, 42130 Konya, Türkiye; 3 Department of Food Processing, Aksaray Technical Sciences Vocational School, Aksaray University, 68100 Aksaray, Türkiye; 4 Departamento de Construcción y Agronomía, Facultad de Ciencias Agrarias y Ambientales, Universidad de Salamanca, 37007 Salamanca, Spain; 5 Estación Tecnológica de La Carne, Instituto Tecnológico Agrario de Castilla y León (ITACyL), Guijuelo, Salamanca, Spain

**Keywords:** Bone properties, Growing quail, Lipid oxidation, Mushroom, Performance

## Abstract

This study was performed to determine the effect of mushroom powder (MP) (*Agaricus bisporus*) supplementation on growing Japanese quail (*Coturnix coturnix japonica*). A total of 300 unsexed 1-day-old Japanese quails with similar body weights (8.38 ± 0.2 g) were randomly assigned to five treatment groups with six replications. Additions of 0, 0.25, 0.50, 0.75, or 1.00% of MP to the basal diet were used to develop the treatment groups. Quails were fed ad libitum for 42 days. At the end of the experiment, 12 quails from each experimental unit were euthanised to determine performance, carcass traits, meat quality, and bone biochemical properties. Results showed that all dietary MP did not negatively affect any performance parameters (*P* > 0.05), while by the third week of life, there was an increase (*P* < 0.05) in body weight and body weight gain in the quails of the 0.75% MP group compared to the control group. Nevertheless, these differences disappeared at the end of the trial (*P* > 0.05). No differences were observed (*P* > 0.05) for any of the studied carcass traits, except for the pancreas weight which decreased (*P* < 0.05) with the addition of high MP (1.00%). Regarding meat quality, all color parameters were affected on the fifth day of sampling (*P* < 0.05) but not on the first day (*P* > 0.05). It was detected that the breast of the quails in group 0.75% MP had the highest L* value and the lowest a* value. While the breast of the 1.00% MP group had the highest *b* value and the lowest pH value. Dietary MP enhanced oxidative stability, reducing malondialdehyde (MDA) value in the breast compared to the control at both sampling points (*P* < 0.01), being more noted on the fifth day of sampling. Bone biomechanical properties (in terms of shear force or shear stress) were improved (*P* < 0.01) with the dietary addition of MP at 0.75% compared to the control. It can be suggested that MP is a secure ingredient in animal feed without negatively affecting performance parameters, carcass traits, or meat quality. Therefore, including an interval of 0.50–0.75% of MP in the diet of growing quails could be a suitable strategy to improve certain parameters such as the meat’s oxidative stability and the bone’s biomechanical parameters. Moreover, the efficacy of MP on performance development would be greater during the first weeks of the quails’ life due to their intestinal conditions at this stage.

## Introduction

The importance of Japanese quail (*Coturnix coturnix japonica*) production has increased in recent years due to its easy handling, short life cycle, and small size (Sarmiento-García et al., 2023a). In addition, quail meat intake has risen in the last few years in countries mainly in Europe and Latin American countries (Vargas-Sánchez et al., 2018), and it can be expected that in close future, quail may be the main bird involved in meat production due to its broad distribution (Sabow et al., 2020). Therefore, researchers should evaluate nutritional strategies to improve quail’s performance and meat quality (Vargas-Sánchez et al., 2018).

Mushrooms are fruiting bodies of fungi known worldwide due to their nutritional, sensorial, and pharmaceutical value (Khan et al., 2019; Kumar et al., 2022). These perceived benefits are associated with the nutritional composition of the mushrooms (Asadi-Dizaji et al., 2017; Khan et al., 2019; Altop et al., 2022). Moreover, mushrooms are a rich source of secondary metabolites and bioactive compounds which could have an important role in promoting health (Giannenas et al., 2010a; Bhushan and Kulshreshtha, 2018; Rangel-Vargas et al., 2021). In addition, it has been shown that certain mushrooms could decrease bone loss and be considered a preventive and/or complementary treatment in patients with osteoporosis (Erjavec et al., 2016). In this sense, including mushrooms in the diet of livestock could be an interesting way to enhance their performance, their health status, and the quality of their products (Giannenas et al., 2010a; Lee et al., 2012; Erjavec et al., 2016; Vargas-Sánchez et al., 2018; Altop et al., 2022).

Currently, more than 11.8 million tons of mushrooms are produced annually worldwide. *Agaricus bisporus* is the most commonly cultivated mushroom (Kumar et al., 2022), and in the last decade, its production has increased (Bhushan and Kulshreshtha, 2018). Nevertheless, about 20% of the mushroom crop is wasted in the manufacturing and packaging processes (Altop et al., 2022; Bhushan and Kulshreshtha, 2018; Kumar et al., 2022). Although reducing food waste needs to be recognized as a critical goal, reintroducing food by-products back into the food supply chain plays an essential role in the application of the circular economy. In this sense, vegetal by-products have been recognized as an important source of bioactive compounds, similar to the initial product (Sarmiento-García et al., 2023b). Considering the beneficial substances and the composition of mushrooms, their byproducts could be a suitable approach to animal feeding contributing to the reuse of by-products and the enhancement of avian health (Bhushan and Kulshreshtha, 2018; Mahfuz et al., 2019; Yang et al., 2021; Altop et al., 2022). Nevertheless, according to Khan et al. (2019) and Guimarães et al. (2014), considerable research has been conducted in the past on the impact of including mushrooms in the animal diet, but few studies are available on the effect of mushrooms on poultry meat quality, and even more scant information is available about these effects on quail. Previous studies proposed that the dosage to improve performance and other parameters of avian production is not clear, and more studies are needed. The current experiment hypothesized that dietary mushrooms will enhance performance development, carcass, meat quality, and bone biomechanical properties in quails. For this purpose, the effects of five graded levels of dietary MP (Agaricus bisporus) on performance, carcass traits, meat quality, and bone biochemical were assessed.

## Materials and methods

### Animal material and experimental diets

Since the study was carried out using farm animals, no particular certification for laboratory animal breeding was demanded. Nevertheless, before its development, the study was approved by the Ethics committee of Aksaray University (2024/1–4). In addition, all procedures have followed the European policy (EPCEU, 2010) for the safety of animals used for scientific research and other scientific purposes, which conform to the ethical standards set out in the 1964 Declaration of Helsinki and its subsequent amendments.

A total of 300 unsexed 1-day-old Japanese quail (*Coturnix coturnix japonica*) with similar body weights (8.38 ± 0.2 g), were randomly allotted to five dietary treatments with six replicates each containing ten quails. The study was conducted using a total randomized design for 6 weeks at a local farm in Eskil, Aksaray, Türkiye (38°1′36, 32°30′45″). All animals were reared under the same controlled environment conditions. The temperature of the experimental house was set at 30 °C during the first week and was gradually reduced to 26 °C during the second week until the end of the experiment, which was controlled by central heating. The lighting schedule was set in 23 h lightness:1 h darkness throughout the experimental period. The quail chicks were housed in identical battery cages (45 cm wide × 30 long cm × 20 cm high) equipped with a feeder and nipple to provide water and feed ad libitum during the whole experiment.

For the experimental treatments, mushrooms that had been discarded for human intake were purchased from a local company in Türkiye (Kurucum Gıda, Ltd. Şti., Isparta, Türkiye). Fresh mushrooms were dried in an oven to reach 60 °C and then they were ground on a 5-mm screen to obtain MP before being incorporated into the feed. Before formulating the diets, the antioxidant properties of mushrooms were estimated using the approach outlined by Singleton and Rossi (1965) for the total phenolic content and the reducing power (%). The chemical composition of the MP was measured as detailed in AOAC (2006) by drying the water to obtain ash (942.05), determining the protein and fat content using the Kjeldahl (990.03) and Soxhlet (2003.06) processes, using the Weende method (978.10) to determine crude fiber and by testing the moisture content (2001.12) at 105 °C for drying. The chemical composition of the MP is given in Table 1.

**Table 1:** Chemical composition, reducing power, flavonoids, and total phenolic compounds of mushroom powder (*n* = 3)

Parameters	Level
Crude protein (%)	27.81
Crude fiber (%)	11.73
Crude fat (%)	0.86
Ash (%)	3.83
DPPH (%)	84.48
Total phenolic content (mg GAE/100 ml)	63.78
Total flavonoid content (mg CE/100 ml)_0_	3.84

*DPPH*, 2,2 diphenyl-1-picrylhydrazyl; *CE*, catechin equivalent; *GAE*, gallic acid equivalent

The experimental design consists of five graded levels (0.00%, 0.25%, 0.50%, 0.75%, or 1.00%) of MP. Considering the low content (< 1%) and the attempt to favorably impact animal product characteristics, MP was evaluated as a feed additive, not a feedstuff. MP was included in diets, which were prepared in mashed form. The basal diet was formulated to achieve the requirements of quails according to the recommendations proposed by the National Research Council (1994). The nutrient composition was analyzed according to the methods described above for MP nutrients. Table 2 describes the ingredients of the basal diet and its chemical composition.

**Table 2:** The ingredients of basal diet and nutrient composition

Ingredient	%	Nutrient composition	%
Corn	49.50	Metabolizable energy (kcal/kg ME)	2902
Soybean meal	43.50	Crude protein	23.97
Sunflower oil	3.20	Crude fiber	3.81
Limestone	1.09	Crude fat	5.43
Dicalcium phosphate	1.86	Calcium	1.01
Salt	0.35	Available phosphorus	0.50
Premix^1^	0.25	Lysine	1.34
DL methionine	0.25	Methionine	0.53
Total	**100.00**	Methionine + cystine	1.00

^1^Premix provides 80 mg manganese (manganese oxide), 60 mg iron (iron carbonate), 5 mg copper (copper sulfate pentahydrate), 1 mg iodine, 0.15 mg selenium, 8800 IU vitamin A (trans-retinol acetate), 2200 IU vitamin D_3_ (cholecalciferol), 11 mg vitamin E (tocopherol), 44 mg nicotinic acid, 8.8 mg Cal-D-Pan, 4.4 mg vitamin B_2_ (riboflavin), 2.5 mg thiamine, 6.6 mg vitamin B_12_ (cyanocobalamin), 1 mg folic acid, 0.11 mg biotin, 220 mg choline to per kg of diet

### Determination of performance parameters

During the course of the study, animal deaths and general conditions were monitored daily. The survival rate was calculated and expressed as a percentage from these data. Upon arrival at the facilities, the animals were weighed individually with a precision balance (± 0.01 g). Subsequently, the weighing was repeated at 3-week intervals (0, 3, 6). Feed intake was estimated by subtracting the quantity supplied from the amount of feed left over in the feeder for each experimental unit as reported by Sarmiento-García et al. (2023a). These values were used to determine the body weight gain and then expressed in g/quail/day for each interval (3-week intervals) and the total study period. The feed conversion ratio (FCR) was determined from feed intake and body weight gain for each period studied (3-week intervals) and for the total period of the experiment.

### Carcass characteristics

At the end of the study (42 days), 12 quails from each experimental unit were randomly selected, weighed, and euthanized by cervical dislocation. After that, they were de-feathered and subsequently weighed to determine carcass weight which was described as the percentage of live weight. The carcass was eviscerated, and the liver, heart, pancreas, and bursa fabricius were individually weighed and expressed as a percentage of body weight.

After 15 min post-mortem, the breast and the thigh (*n* = 60) were separated and weighed, and then they were expressed as the percentage of body weight. Then, the breast (*Pectoralis major muscle*) was kept at − 4 °C using ice boxes and immediately sent to the laboratory (Selcuk University, Faculty of Agriculture, Meat and Meat Products Research Laboratory, Türkiye) for the rest of the analysis.

### Determination of meat quality traits

Color and pH values were evaluated in the left breast (*n* = 60) without breast skin. In both cases, the procedure was repeated thrice per sample, and these values were used to calculate the average value. Both procedures were measured at 1 h (1d) and 5 days (5d) after animals were euthanized. Samples for the analysis on the fifth day were kept in a chilling room at − 4 °C for 5 days. Before the analysis, skin breasts were separated, and breast muscles were kept at 4 °C for 1 h to allow oxygenation of the myoglobin.

The pH values were determined using a pH meter (Testo 205 pH-Temperatur-Messgerat, AG Postfach 1140, 79849, Lenzkirch) equipped with a penetration electrode. The meat color was determined on the external surface of the *pectoralis* muscle using a colorimeter (CR-200, MinoltaCo., Osaka, Japan). The color measurement was made perpendicular to the sample surface avoiding fat or connective tissue. L* (lightness), a* (redness), and b* (yellowness) were estimated using an observer of 10° and the illuminant D65 in the CIELab space.

To quantify the oxidative stability of breast samples, the thiobarbituric acid reactive substance (TBARS) test was performed in duplicate. As with pH and color, the TBARS method was performed twice in the experiment (1d and 5d). The modified method described by Sarmiento-García et al. (2021) was carried out for each sample. Ten grams per sample were weighed and mixed with 50 mL of distilled water using an UltraTurrax (IKA, USA). From the resulting mixture, 1 ml was taken and mixed in a tube with 50 µL of butylated hydroxyanisole (7.2%) and 2 mL of a solution containing 0.375% thiobarbituric acid (TBA) and15% trichloroacetic acid (TCA). Tubes were placed in a water bath for 30 min to develop a pink color related to the malondialdehyde (MDA) content of the sample. After that, the mixture was cooled using tap water and centrifugated (5 min, 2000 rpm). The supernatant was used to determine the absorbance values using a spectrophotometer (PerkinElmer, USA) at a 530 nm wavelength. The blank containing 1 mL of TCA extraction solution and 1 mL of TBA solution was employed as a benchmark for the spectrophotometric measurement (Perkin Elmer, USA). TBA value was expressed as µmol MDA/kg meat using the Eq. (1) described by Kilic and Richards (2003).

1 $$TBARS =\frac{\left(\frac{absorbance}{k}x\frac{2}{1000}\right)x6.8 }{sample\;weight}X 1000$$

### Evaluation of bone biomechanical parameters

The procedures described below were performed according to those reported by Gül et al. (2022). For this purpose, the right tibia (*n* = 60) was taken, and it was cleaned of its associated tissue and kept at − 20 °C before the analysis. Then, before the analysis, the samples were placed in a controlled air environment at room temperature for 6 h.

The cortical bone thickness of the tibia was determined using digital calipers (0.001 mm precision) based on two points from the central axis of the fractured tibia, which were employed to assess the mechanical properties. From these values, the bone diameter (mm) was determined according to the following Eq. (2).

2 $$Bone\;diameter= {(\frac{\left(diameter\;of\;tibia \right)}{2})}^{2}- ({\frac{cavity\;diameter\;of\;tibia }{2})}^{2}$$

From the value obtained in Eq. 1, the value of the cortical bone cross-sectional area (mm^2^) was calculated according to Eq. 3.

3 $$\mathrm{cortical\;bone\;cross}-\mathrm{sectional\;area}= \pi \frac{bone\;diameter}{2}$$

The tibia mechanical traits were determined by the load-deformation curve generated using the Universal Testing Machine (Instron 1122) and the Test Works 4 software package (version 4.02; MTS System Corporation, Eden Prairie, MN). The cross-head speed was 5 mm/min. Tibial shear tests were performed using a double-shear block apparatus. A shear force was exerted on a 6.35 mm (0.25 in.) section at the center of the diaphysis. These tests enabled the assessment of the value of ultimate shear force (N) which would be used to estimate the shear stress (N/mm^2^) using the following Eq. (4).

4 $$shear\;stress=\frac{shear\;force}{cortical\;bone\;cross-sectional\;area}$$

### Statistical analysis

Results were statistically evaluated for performance, carcass traits, meat quality, and bone biomechanical parameters. All available data were examined by one-way ANOVA with the SPSS 22.0 software package (SPSS Inc., Chicago, IL, USA). A *P*-value less than 0.05 was taken as statistically significant, while a *P*-value less than 0.10 was set as a trend. Orthogonal polynomial contrasts for determining the significance of linear and quadratic models were used. It was tested to describe the response of the variable to an increasingly high level of MP.

## Results

### Performance parameters

The effect of adding different levels of MP to growing quail diets on performance parameters is demonstrated in Table 3. According to the randomized design of the experiment, no significant differences (*P* > 0.05) were observed for the body weight at the first measurement between experimental treatments. At day 21 of age (3 weeks), the body weight of quails in the 0.75% group was significantly higher than those obtained in the 0% and 0.25% groups (*P* < 0.05). Nevertheless, these differences disappeared in the sixth week (*P* > 0.05). Body weight gain demonstrated similar trends. The quails from the 0.75% group had a body weight gain greater than the control and 0.25% groups (*P* < 0.05) for 0–3-week intervals. The quails from the 0.25% group did not statistically (*P* > 0.05) differ from the quails of the control group; nevertheless, it was numerically higher. The rest of the study intervals (including the total study period) revealed no differences between the experimental groups in terms of body weight gain.

**Table 3:** Effect of dietary addition of mushroom powder on growing quail performance (*n* = 300)

Parameters	Mushroom powder level (%)					S.E.M	*P-*value		
	0	0.25	0.50	0.75	1.00		Anova	*L*	*Q*
Body weight (g)									
Hatching	8.37	8.38	8.38	8.38	8.40	0.053	0.995	0.693	1.000
3. week	122.26^b^	122.80^b^	123.48^ab^	128.55^a^	126.40^ab^	1.310	0.010	0.002	0.842
6. week	222.18	225.88	222.60	228.09	224.04	3.090	0.647	0.549	0.566
Body weight gain (g)									
0–3. week	113.89^b^	114.42^b^	115.09^ab^	120.17^a^	118.00^ab^	1.299	0.009	0.002	0.841
3–6. week	99.93	103.08	99.13	99.54	97.64	2.963	0.769	0.395	0.609
Total (0–6. week)	213.82	217.50	214.22	219.71	215.64	3.084	0.646	0.553	0.565
Feed intake (g/quail)									
0–3. week	234.20	238.74	236.69	243.63	242.33	2.430	0.062	0.011	0.768
3–6. week	539.30	550.10	526.00	550.94	525.30	13.642	0.516	0.532	0.644
Total (0–6. week)	773.50	788.90	762.70	794.57	767.60	14.110	0.455	0.891	0.619
Feed conversion ratio									
0–3. week	2.06	2.09	2.06	2.03	2.05	0.023	0.529	0.385	0.918
3–6. week	5.40	5.34	5.34	5.55	5.39	0.130	0.793	0.655	0.994
Total (0–6. week)	3.62	3.63	3.56	3.62	3.56	0.047	0.733	0.430	0.928
Survival rate (%)	88.33	95.00	96.67	95.00	90.00	4.978	0.717	0.834	0.165

*S.E.M.*, standard error means; *L*, linear effect; *Q*, quadratic effect

^a,^^b^Means with different superscripts in the same row were significantly different (*P* < 0.05)

Feed intake was not affected by any of the intervals studied depending on the experimental group (*P* > 0.05). However, there is a linear increase in feed intake in the 0–3-week interval (*P* < 0.05). Feed intake of quails was maximum at 0.75% MP level. For the feed conversion ratio (FCR), no differences (*P* > 0.05) were described at any of the intervals studied. It is interesting to highlight that the control group and the 0.75% group had the same FCR value during the entire study period (0–6 weeks). No differences (*P* > 0.05) were observed in the survival rate between the different experimental groups. However, the numerical value of this parameter was lower in the control group than in the groups that had received MP in the diet.

### Carcass characteristics

The effect of dietary MP on slaughtering and carcass parameters is described in Table 4. In growing quails, adding the MP to the diet had no statistical effect (*P* > 0.05) on carcass yield, or relative thigh or breast weights, showing similar values to the control.

**Table 4:** Effect of dietary addition of mushroom powder on carcass weight (*n* = 60) in growing quails

Parameters	Mushroom powder level (%)					S.E.M.^*^	*P-*value		
	0	0.25	0.50	0.75	1.00		Anova	*L*	*Q*
Carcass (%)	61.90	60.59	62.27	60.97	59.93	0.627	0.084	0.085	0.305
Thigh (%)	21.29	20.83	21.43	20.42	21.01	0.569	0.742	0.596	0.815
Breast (%)	31.92	29.81	31.75	28.73	28.87	1.263	0.238	0.084	0.926

All parameter values are expressed as a percentage of body weight (%). *S.E.M.*, standard error means; *L*, linear effect; *Q*, quadratic effect

According to Table 5, it is shown that liver, heart, and bursa fabricius weights of quails presented similar values that no differ among experimental treatments (*P* > 0.05). However, the pancreas weight of the control group showed significantly increased values (*P* < 0.05) compared to the 1.00 group (0.23 vs. 0.16%), while the rest of the experimental groups showed intermediate values.

**Table 5:** Effect of dietary addition of mushroom powder on visceral weights (*n* = 60) in growing quails

Relative weights	Mushroom powder level (%)					S.E.M	*P-*value		
	0	0.25	0.50	0.75	1.00		Anova	*L*	*Q*
Liver (%)	1.95	2.04	1.81	1.89	1.93	0.112	0.685	0.584	0.619
Heart (%)	0.86	0.84	0.83	0.87	0.90	0.039	0.713	0.368	0.303
Pancreas (%)	0.23^a^	0.19^ab^	0.18^ab^	0.18^ab^	0.16^b^	0.013	0.038	0.003	0.454
Bursa fabricius (%)	0.09	0.09	0.09	0.08	0.09	0.012	0.991	0.846	0.724

All parameter values are expressed as a percentage of body weight (%). *S.E.M.*, standard error means; *L*, linear effect; *Q*, quadratic effect. ^a,b^Means with different superscripts in the same row were significantly different (*P* < 0.05)

### Meat quality traits

Table 6 shows the effects of dietary MP on breast meat quality characteristics after 1 h of slaughter (1d) and after 5 days (5d) of storage (− 4 °C). The L*, a*, and b* values of quail breast meat were not significantly (*P* > 0.05) affected by MP dietary at 1 day (1d). However, on day 5 of storage (5d), all color parameters assessed in quail breasts exhibited significant differences between the experimental groups. The breast of quails that had received 0.75% MP showed an increase (*P* < 0.01) in the lightness value (L*) of the breast. This value was similar to those obtained in the breast of the 1.00% group and higher than the value of the breast from the rest of the experimental groups. For the redness value (a*), a decrease in the a* value (*P* < 0.05) was observed in the breast of the 0.75% group compared to the breast of the control group. As can be seen in Table 6, the yellowness value (b*) value of the breast from the 0.50% group was significantly lower (*P* < 0.01) than the values obtained for the breasts of the control and 1.00% groups owing intermediate values in the breast for the rest of the groups.

**Table 6:** Effect of dietary addition of mushroom powder on some quality traits in breast meat (*n* = 60) at day 1 (d1) and 5 (5d) of storage conditions

Parameters	Mushroom powder level (%)					S.E.M	*P-*value		
	0	0.25	0.50	0.75	1.00		Anova	*L*	*Q*
L^*^									
1d	35.50	37.05	36.70	38.16	36.71	1.077	0.551	0.312	0.309
5d	38.47^bc^	38.09^bc^	36.24^c^	40.86^a^	39.51^ab^	0.540	< 0.001	0.009	0.034
a^*^									
1d	18.93	19.68	19.05	17.20	18.28	0.920	0.403	0.206	0.870
5d	18.04^a^	17.36^ab^	16.93^ab^	14.34^b^	17.34^ab^	0.744	0.017	0.072	0.074
b^*^									
1d	8.37	9.19	8.22	8.59	9.21	0.531	0.568	0.530	0.637
5d	12.03^a^	11.24^ab^	8.10^b^	8.71^ab^	11.76^a^	0.852	0.006	0.263	0.001
pH									
1d	5.87^a^	5.79^ab^	5.78^ab^	5.77^ab^	5.75^b^	0.024	0.018	0.003	0.164
5d	5.81^ab^	5.85^ab^	5.87^a^	5.85^ab^	5.76^b^	0.024	0.029	0.176	0.003
MDA value									
1d	0.66^a^	0.60^b^	0.56^b^	0.57^b^	0.56^b^	0.013	< 0.001	< 0.001	0.004
5d	1.20^a^	0.97^b^	0.84^b^	0.79^b^	0.47^c^	0.047	< 0.001	< 0.001	0.595

MDA values are expressed as µmol/kg. *S.E.M.*, standard error means. ^a,b,c^Means with different superscripts in the same row were significantly different (*P* < 0.05). *L*, linear effect; *Q*, quadratic effect

The findings of the current study for the pH values (Table 6) exhibited that on the first day (1d), the pH value of the breast meat of quails fed with diets containing 1.00% MP was significantly lower than the control group (*P* < 0.05). On day 5 (5d), trends partially similar to those reported in 1d were described; as can be seen in Table 6, the lowest pH value was recorded in the breast of group 1.00%, while here the highest pH value was observed in the breast of group 0.50% (*P* > 0.05).

Table 6 demonstrates that all quails that received MP in the diet (0.25, 0.50, 0.75, and 1.00%) significantly reduced the MDA values of breast meat on the first day (1d) compared to the breast of the control group (*P* < 0.01). This decline became even more obvious on the fifth day of storage (5d). The lowest value of MDA (*P* < 0.01) was recorded in the breast of the 1.00% group, followed by the rest of the groups that had received MP in the diet (0.25, 0.50, and 0.75%). In all cases, the breast MDA value was significantly higher (*P* < 0.01) than that observed in the control group.

### Bone biomechanical properties

The effect of MP dietary on the bone biomechanical properties of quails is demonstrated in Table 7. No differences (*P* > 0.05) were described between experimental groups for the cortical bone thickness, bone diameter, and cortical bone cross-section showing all groups’ values similar to the control. However, differences were recorded for the rest of the bone biochemical properties studied. It is important to point out, that for shear force and the shear stress parameters, the highest (*P* < 0.01) values were recorded in the bone of the 0.75% group, being higher than the value of the control group. A higher value was recorded in shear force for the bones of the 0.75% group than the bones of the control, 0.25%, and 1.00% groups (*P* < 0.01). Similar trends were described for the shear stress value. In this case, the highest value was also recorded in the bone of the 0.75% group, being higher than those obtained in the 1.00% group, which was also higher than the value from the control group (*P* < 0.01).

**Table 7:** Effect of dietary addition of mushroom powder on bone biomechanical properties of quails (*n* = 60)

Parameters	Mushroom powder (%)					S.E.M	*P-*value		
	0	0.25	0.50	0.75	1.00		Anova	*L*	*Q*
Cortical bone thickness (mm)	0.56	0.53	0.51	0.55	0.57	0.032	0.734	0.674	0.229
Bone diameter (mm)	2.92	2.85	2.74	2.89	2.89	0.059	0.271	0.940	0.088
Cortical bone cross-sectional area (mm^2^)	4.12	3.88	3.59	4.02	4.11	0.194	0.299	0.856	0.066
Shear force (N)	126^b^	144^b^	159^ab^	191^a^	140^b^	10.8	0.003	0.038	0.006
Shear stress (N/mm^2^)	30.9^c^	37.5^abc^	44.8^ab^	47.9^a^	34.4^bc^	3.07	0.003	0.086	0.001

*S.E.M.*, standard error means. ^a,b,c^Means with different superscripts in the same row were significantly different (*P* < 0.05). *L*, linear effect; *Q*, quadratic effect

## Discussion

### Performance

*A. bisphorum* (AB) is considered one of the most promising edible mushrooms in animal nutrition due to its nutritional composition, which also includes antioxidant substances such as vitamins, minerals, and polyphenols (Kumar et al., 2022) that may provide favorable effects on animal health (Asadi-Dizaji et al., 2014; Altop et al., 2022). In the current research, MP addition to the growing quails’ diets showed differences between experimental groups for performance parameters. Regarding the body weight, at 3 weeks, the quails in the 0.75% group showed a higher body weight than the quails that had not received PM in the diet or that had received it in low doses (0.25%), leading to the same differences between these groups in body weight gain values for the 0–3-week interval. Similar findings have been described by previous authors. Giannenas et al. (2014) described an enhanced performance of broiler chickens when AB was added to the diet (1% and 2%) similar to those described for male quails and broilers (2%) by Asadi-Dizaji et al. (2014) and Toghyani et al. (2012) respectively. Hassan et al. (2020) described that mushroom addition (*Pleurotus osteratus*) improves the broiler body weight when it was added at 1% which is in accordance with Kavyani et al. (2012), while Guimarães et al. (2014) recorded the best performance parameters with 1.6 g mushroom/kg diet. The inclusion of mushrooms in the poultry diet could improve body weight and body weight gain due to an enhancement in the avian gut including the development of villi and the decreased counts of *Bacteroides* spp., *Escherichia coli*, and *Enterococci* due to their immune-stimulatory effects and nutritional composition, especially b-glucan substances (Khan et al., 2019; Mahfuz et al., 2019). They might have influenced the balance of the intestinal tract microflora by acting as growth promoters and prebiotics (Khan et al., 2019). Moreover, including AB in the diet of avian species could control and limit the growth and colonization of pathogenic species such as *Coccidian* or *Eimeria* spp. affecting not only the growth delay of chicks but also their survival (Kavyani et al., 2012). This may have occurred in this study, since although there were no significant differences in the survival rate, the control group showed a numerically lower value than the rest of the groups that had received MP in the diet. This also confirms that, even at high doses, the inclusion of PM in the quail diet is a safe option which is consistent with those described by Martinez et al. (2022).

According to the current findings, no clear relationship exists between the increase in the dietary dose of mushrooms and the improvement of productive parameters. The quails from the high levels of dietary MP (1.00%) group showed similar performance parameters compared to those obtained in the rest of the control and experimental groups. In accordance with that, Hassan et al. (2020) described that the addition of mushrooms at levels of 2% showed similar body weight to the control group which is in accordance with Guimarães et al. (2014). It could be attributed to the high fiber total content of AB (Bhushan and Kulshreshtha, 2018). Higher levels of crude fiber could lead to a reduction of nutrient utilization in quails affecting the body weight. Nevertheless, the differences observed in performance parameters between previous studies concerning the optimal dose of mushroom may be due to the species and the part of the mushroom involved, which determines its composition, as well as the experimental bird and its productive stage (Mahfuz et al., 2019). In addition, Kavyani et al. (2012) pointed out that beneficial effects on performance parameters of MP would be more pronounced under stress conditions (such as infection) or zootechnical conditions are not adequate (Martinez et al., 2022) being responsible for the differences between the studies consulted.

In addition, it is important to note that differences in body weight were only recorded in the 3–6-week interval, with similar body weight values at the end of the study between the experimental groups. Similar findings were reported by previous authors (Kavyani et al., 2012; Guimarães et al., 2014). During the first weeks of the life of the birds, the large intestine contains different active bacteria that participate in the hydrolysis of carbohydrates resulting in a decrease in intestinal pH and an increase in the production of short-chain fatty acids which can inhibit dangerous bacteria and cause the stimulation of beneficial bacteria. Consequently, the inclusion in the diet of ingredients rich in polysaccharides, such as AB, that stimulate this process would be of greater importance in the early life stages of poultry, leading to a more pronounced improvement in performance parameters at this stage. Whereas in older birds, nutrition may have decreased with age, and they may have a more developed digestive tract (Khan et al., 2019). Therefore, the most noticeable effect of the mushroom diet would occur in the early weeks, as observed in this study.

Regarding FCR and feed intake, controversies exist among available studies. As reported in the current research, previous authors (Toghyani et al., 2012; Guimarães et al., 2014; Martinez et al., 2022) did not find differences in feed intake and FCR in the entire experiment between experimental groups which had received mushrooms in the diet. On the other hand, Asadi-Dizaji et al. (2014) described an improvement in the value of FCR when MP was added to the diet at a 2% level, which is similar to those reported by Guimarães et al. (2014) and Kavyani et al. (2012). These authors described that including mushrooms in avian diets leads to more effective utilization of dietary nutrients in the intestinal tract. A more beneficial microbiota population in the intestinal tract of the avian which had received mushrooms in the diet may result in a greater utilization of feed. These findings are partially in accordance with the results of the current study. Although the quails from the 0.50% group showed similar body weight to the rest of the groups at the end of the experiment, this group recorded the lowest numerical value of FCR. However, the differences between the groups were not significant, so further studies could be useful. Based on the above results, it could be concluded that the inclusion of MP in the diet of growing quails would be advisable during the first weeks of life with levels between 0.50 and 1.00%.

### Carcass characteristics

No differences were found for the carcass yield and relative thigh rate and breast between experimental groups. Nevertheless, a trend was found for the carcass yield showing the highest values in the carcass from the 0.75% group. This finding is to be expected, considering that the numerical value of the live weight of these animals was higher than that of the rest of the quails from the groups studied (Sarmiento-García et al., 2023a). The current results agree with the previous reports which described the addition of mushrooms to the diet did not affect the carcass yield, leg, and breast ratio in avian species (Asadi-Dizaji et al., 2014; Vargas-Sánchez et al., 2018) and broilers (Daneshmand et al., 2011; Toghyani et al., 2012; Kavyani et al., 2012; Guimarães et al., 2014; Kidane et al., 2017; Hassan et al., 2020; Altop et al., 2022). Contratry, Martinez et al. (2022), found that 2.5 g/kg MP improved both carcass yield and relative breast weight whereas thigh meat was unaffected. These authors attributed the results to an improvement in intestinal health and nutrient absorption (especially in amino acids), which led to enhanced muscle synthesis. However, it is not clear. Moreover, these authors reported that these improvements could be more evident when the use of mushrooms in the diet is prolonged. Nevertheless, they concluded that more studies are needed to understand these effects.

The liver, heart, and bursa fabricius weights of growing quails were not affected by the addition of MP to the quails’ diet which is partially in agreement with the results described by previous authors (Toghyani et al., 2012; Fard et al., 2014; Abro et al., 2016; Fanhani et al., 2016; Hassan et al., 2020; Altop et al., 2022). Aguilar et al. (2011) pointed out that an elevation in the relative weight of lymphoid organs in healthy birds may not always be associated with an enhancement of immune activity. In the current research, a decrease in pancreas weight was described, reaching the lowest value in the pancreas from the 1.00% group. These results differ from the previous studies which found that the inclusion of mushrooms into the diet did not affect the pancreas weight of broilers (Toghyani et al., 2012; Altop et al., 2022), but the current findings differ from those of the earlier studies. In the current research, it can be hypothesized that the lower pancreatic weight in groups consuming feeds containing 1.00% MP may be due to increased efficiency and digestibility due to the active ingredients in the MP. Aderibigbe et al. (2020) reported that pancreatic weight decreased with the use of additives such as enzymes that improve nutrient digestion. Since pancreatic enzymes (especially pancreatic enzyme release) were not analyzed in the present study, further research is required to determine the effect of MP on pancreatic function. In this way, the decrease in pancreatic weight may be understood.

### Meat quality traits

Color has been described as one of the most important meat qualities, playing a pivotal role in purchase decisions (Vieira et al., 2021). In the current research, the breast color parameters were similar to those reported by previous authors (Vargas-Sánchez et al., 2022; Sarmiento et al., 2023a) for growing quails. According to the value obtained, this meat could be described as dark meat which is linked to values of L* < 50.0, a* > 4.5, and b* < 10.0 (Vargas-Sánchez et al., 2018). Regarding MP inclusion, no differences were described between the breasts from the different experimental groups on the first day of sampling (1d). However, differences were described on day fifth (5d). The values recorded in the breast from the 0.75% group had higher L* and lower a* values than the control group, while the lowest b* value was recorded in the breast from the 0.50% group. There does not seem to be clear agreement about the effect of mushroom addition to poultry diets on breast color. Vargas-Sánchez et al. (2018) reported that the addition of 10 and 20 g/kg MP to the growing quails’ diet decreased L* and increased the a* values of the meat samples on the 15th day of storage. Similarly, Martinez et al. (2022) observed that 2.5 g/kg of MP decreased the L* and increased the a* values of the breast meat of broilers. On the other hand, Altop et al. (2022) found that the L* value increased, while it decreased in breast meats of broilers fed with diets containing 20 g/kg mushroom. Mushrooms are a good source of bioactive compounds that could lead to changes in meat color (Kumar et al., 2022). The differences between the reviewed studies may be because the extraction and processing of mushrooms cause changes in their composition.

In the current research, a lower pH value was recorded on the first sampling day in the breast of the 1.00% group than in the control group (5.75 vs. 5.87). However, on the fifth sampling day, the pH value was higher in the breast from the 0.50% group than in the 1.00% group (5.87 vs. 5.76). In this sense, the inclusion of MP at a 1.00% dose in the diet of growing quails would be the most optimal dose to decrease the pH value, which is related to meat tenderness (Guimarães et al., 2014). Nevertheless, in all cases, the average pH value of the breast from experimental groups varied from 5.75 to 5.87, and it was close to the values reported in other studies for growing quails (Vargas-Sánchez et al., 2018; Sarmiento-Garcia et al., 2023a), which describe normal values of pH ranging 5.8 to 6.3. The values obtained in the current study suggest good animal welfare standards, which would lead to a decrease in pre-slaughter stress and, therefore, a reduction in muscle glycogen use as described by Vargas-Sánchez et al. (2018). The effect of dietary mushrooms on the pH values of the breast is not clear, and there are some that are controversial. For example, Vargas-Sánchez et al. (2018) reported that the dietary addition of 20 g/kg mushroom decreased the pH values of breast meat on the fifth day of storage, which is similar to those described by Guimarães et al. (2014) (using levels at 0.4, 0.8, 1.6, and 2.0 g/kg of mushrooms). However, Altop et al. (2022) noted that the administration of mushrooms to the diet increased the pH of breast meat in broilers. As described above, pre-slaughter factors appear to play a more important role in meat pH than dietary treatments. Conditions that stress the animal, such as heat stress during transport and handling, restrict glycolysis in muscle after slaughter raising the pH of the meat (Herawati and Marjuki, 2011). Therefore, further studies are required to confirm whether the effect of variations in pH value can be attributed to the inclusion of mushrooms in the diet.

Malondialdehyde (MDA), which is the last product of lipid peroxidation, can be used to estimate the degree of lipid oxidation (Gökmen et al., 2022). Lipid peroxidation affects meat quality, worsening its attributes such as color, flavor, texture, and nutritional content (Giannenas et al., 2010b). In the current study, including PM in the diet of growing quail enhanced the antioxidant capacity of the meat, as evidenced by the reduction in MDA value. This fact is even clearer on the fifth day of storage (5d), where higher levels of PM in the diet (1.00%) depressed MDA values compared to the control group. *A. bisporus* is an edible mushroom rich in phenolic compounds (Altop et al., 2022) which play a pivotal role in the antioxidant activity. Low molecular weight phenolic compounds, although they have low liquid uptake in the intestine, achieve the bloodstream and potentiate antioxidant activity in animals (Giannenas et al., 2010a). Moreover, as shown in this study, the antioxidant activity seems to be dose-dependent, and the protective effect of *A. bisporus* results from the capacity of polysaccharide and polyphenol fractions to stabilize free radicals by electron transfer (Fanhani et al., 2016). These compounds can act as inhibitors of MDA by accumulating and transferring this compound to the meat. (Vargas-Sánchez et al., 2018). The results obtained in the current research are in agreement with previous authors who showed the effectiveness of mushrooms dietary to alleviate the adverse effects of lipid oxidation in meat (Giannenas et al., 2010b, 2011; Vargas-Sánchez et al., 2018; Hassan et al., 2020). Nevertheless, the MDA values of the breast were raised for all groups over time (1 to 5 days), indicating that lipid peroxidation occurred from day 1. This fact could be due to the low dose used in their diet, which would allow a lower value than that of the control group, but which would increase with time. Similar findings were described by Fanhani et al. (2016). However, it is important to point out that the value reached on the fifth sampling day was higher in the breast of the control group than in the rest of the experimental treatments which is in agreement with those described by Giannenas et al. (2010a, 2011) and reasserts the antioxidant efficacy of the AB.

### Bone biomechanical properties

Leg problems in poultry cause significant economic losses, such as slower growth due to the inability of birds to feed, increased mortality, deterioration of carcass quality, and increased deaths during transport to slaughter (Olgun and Aygun, 2016). Therefore, finding ways to solve this problem seems to be of great importance in avian production. The main factors affecting bone mineralization and preventing osteoporosis include calcium, phosphorus, and vitamin D, which catalyze its homeostasis (Perez-Lopez et al., 2011; Schnatz et al., 2011; Olgun and Aygun, 2016). The addition of MP to growing quail diets did not significantly affect the cortical bone thickness, bone diameter, and cortical bone cross-sectional. Nevertheless, the supplementation of 0.75% MP to the diet significantly increased the shear force value of the bone compared to the control, 0.25, and 1.00 groups. Related patterns are reported for shear stress value. Mushrooms are a rich source of ergosterol which is a precursor of vitamin D, calcium, and other bioactive compounds which have immunomodulatory activity (Lindequist and Haertel, 2021). These results suggest that the optimal dose of MP would be between 0.50 and 0.75% and that higher or lower doses would not have an improvement in bone parameters. High doses (1%) of MP are probably related to lower absorption and digestibility of vitamin D and calcium, as has been described for other parameters studied at this dose. To our knowledge, there is no research examining the effect of MP or mushroom products on the biomechanical properties of bone. Partially in accordance with the current results, previous authors have described that including aromatic plants or essential oils, which are rich in calcium and bioactive substances, in animals’ diets could enhance the structural properties of the bone by improving the absorption of minerals (Zhou et al., 2009; Kwiecien et al., 2014; Olgun, 2016; Rehman et al., 2018; Gül et al., 2022; Liu et al., 2022). Further studies on animals are needed to investigate the potential effect of mushrooms on bone biomechanical properties; in addition, the compounds responsible for this potential effect need to be identified.

## Conclusion

The *Agaricus bisporus* is the most commonly cultivated mushroom, and its production leads to the production of important amounts of waste, which could be used as animal feed. According to these results, supplementation of growing quail diets with a range of 0.50–0.75% MP could improve meat oxidation stability and some parameters of quail bone strength, without adverse effects on performance parameters or carcass traits. Likewise, a favorable effect on the productive development of quails was observed with the inclusion of MP at a dose of 0.75% during the first weeks of life (interval 0–3). This fact supports that due to the physiological characteristics of the birds, the MP would have a greater immunomodulatory and protective effect in the first weeks of life linked to the gastrointestinal status at this stage. Therefore, it can be concluded that the inclusion of MP as an additive would be advisable in quail production.

## Data Availability

The authors declare that all the data and materials used in this study comply with field standards and are available on demand.
